# Accelerated Flowering and Differential Florigen Gene Expression of Seagrass *Zostera marina* Under Experimental Warming

**DOI:** 10.1002/ece3.72942

**Published:** 2026-01-14

**Authors:** Christine T. Nolan, Ian T. McBride, Niyah Reid, Sylvia Yang, Takato Imaizumi, Jennifer L. Ruesink, Jeffrey L. Gaeckle

**Affiliations:** ^1^ Department of Biology University of Washington Seattle Washington USA; ^2^ Shannon Point Marine Center Western Washington University Anacortes Washington USA; ^3^ Padilla Bay National Estuarine Research Reserve, Washington State Department of Ecology Mount Vernon Washington USA; ^4^ Washington State Department of Natural Resources Nearshore Habitat Program Olympia Washington USA

**Keywords:** angiosperm, annual life history, ecotype, eelgrass, florigen, gene expression, seagrass flowering, *Zostera marina*

## Abstract

Flowering is an important trait for the resilience of marine angiosperms (seagrasses) as they face rising seawater temperatures, more frequent extreme weather events, and anthropogenic disturbances. Using the seagrass *Zostera marina* , we applied a common garden approach to experimentally test how flowering and its underlying molecular mechanisms responded to elevated water temperature (+3°C). We focused on developmental and reproductive traits paired with florigen gene expression to gain insight into the molecular mechanism underpinning differences in flowering responses. We compared annual seedlings from two source populations (Willapa Bay along the coast and Padilla Bay in the Salish Sea, Washington, USA) to understand natural variation not only in morphological and reproductive traits but also in the gene–environment interactions governing flowering onset. At the individual and population levels, annual seedlings in the +3°C heated treatment produced more spathes and accelerated the development of inflorescences, so seeds dispersed sooner. Seedlings from Padilla Bay flowered at greater rates and earlier than those from Willapa Bay, and these differences were exaggerated by the +3°C heated treatment. A predicted repressor of flowering onset, *ZmaFT9*, was expressed at lower levels in the shoots grown in the +3°C heated treatment, and even more so in the Padilla Bay population, which flowered earlier than the Willapa Bay population. For two predicted floral activators, *ZmaFT2* and *ZmaFT4*, expression increased throughout the summer regardless of population and showed no response to the temperature treatment. *ZmaTFL1a*, a gene predicted to be involved with downstream flowering processes, showed no significant response to the temperature treatment. Together, these results support a key role for antiflorigen (*ZmaFT9*) in the molecular control of flowering in *Z. marina* , and *ZmaFT9* expression contributes to the temperature‐based response of the timing of flowering onset. Impacts of elevated seawater temperature on flowering timing and spathe production, with different responses by population, have potential consequences for seed yield and variation in meadow resilience.

## Graphical abstract

Flowering is an important trait for the resilience of seagrasses. Using a native seagrass, *Zostera marina*, we applied a common garden approach to experimentally test how flowering and its underlying molecular mechanisms responded to elevated water temperature (+3°C). Our results indicate the importance of a temperature‐sensitive antiflorigen gene (*ZmaFT9*) that contributes to the temperature‐based response of timing of flowering onset.

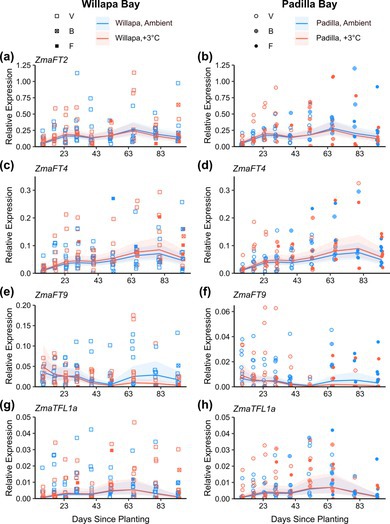

## 1 Introduction

Seagrasses are marine angiosperms that serve as foundation species in coastal ecosystems. They provide key ecosystem services such as sediment stabilization, nutrient cycling, and habitat and feeding grounds for fish, invertebrates, and waterfowl (Orth et al. 2006). Flowering and seed production contribute to genetic diversity (Kendrick et al. 2012) and support recovery following disturbance and seagrass resiliency (O'Brien et al. 2018; Orth et al. 2006).

Temperature has been frequently identified as an important driver of flowering in seagrass, among other environmental factors (Lekammudiyanse et al. 2024). However, the response of seagrasses to elevated temperatures has varied by flowering trait, species, geographic location, and temperature regime, and suggests a need for a mechanistic understanding of flowering. Seasonally elevated temperatures have been observed to both increase or decrease flowering production (De Cock 1980; Diaz‐Almela et al. 2007; Qin et al. 2020; Thom et al. 2003) and advance flowering timing and maturation of seeds (Blok et al. 2018; Sawall et al. 2021). Thus, elucidating the effect of environmental variation, specifically temperature, on the mechanism of flowering is important for understanding the ecological resilience of seagrasses in a changing environment.

The onset of flowering processes in angiosperms is largely regulated and dictated by the phosphatidylethanolamine‐binding protein (PEBP) gene family. One of these genes, *FLOWERING LOCUS T* (*FT*), is the primary activator of flowering, with expression cued in response to environmental inputs and the plant's circadian clock (external coincidence model; de Montaigu et al. 2010). Other genes in the PEBP family can act as repressors of flowering (Kobayashi et al. 1999). The *FT* pathway and the *PEBP* gene family are highly conserved across flowering plants (Bennett and Dixon 2021; Pin and Nilsson 2012; Wickland and Hanzawa 2015), with recent work extending to aquatic and marine plants (Nolan et al. 2025; Yoshida et al. 2021). In the seagrass *Zostera marina* (eelgrass), four of the 14 known PEBP genes have been characterized functionally to influence the floral transition. Two genes, *ZmaFT2* and *ZmaFT4*, cluster within the *FT* clade and are likely activators (Nolan et al. 2025). A third gene in the *FT* clade, *ZmaFT9*, is likely a repressor and appears to be the major determinant of flowering in eelgrass. A fourth gene clusters in the *TFL* clade, within which *TERMINAL FLOWER 1* (*TFL1*) is the main repressor in *Arabidopsis thaliana* ; however, *ZmaTFL1a* in eelgrass relates to shoot determinacy and architecture and not to flowering onset.

Warm temperatures are associated with earlier flowering in eelgrass (Blok et al. 2018), as occurs in many angiosperms. Mechanistically, in *Arabidopsis*, *FT* expression is upregulated and more easily trafficked as a signal in increased temperatures, which results in earlier flowering (Balasubramanian et al. 2006; Blázquez et al. 2003; Kinmonth‐Schultz et al. 2016; Susila et al. 2021). Yet earlier flowering time associated with warmer temperatures leads to lower seed yields in *Zea mays* (Craufurd and Wheeler 2009). In the seagrass *Posidonia*, heat stress led to activation of regulatory genes for flowering and stress tolerance (Marín‐Guirao et al. 2019). Still, much remains to be explored in seagrasses regarding temperature effects on flowering onset from the level of florigen (*FT/TFL1*) gene expression through variation in successful seed production.

In this study, flowering traits are examined in the annual ecotype of *Z. marina* . Annual ecotypes are uncommon but widespread across the northern hemisphere range of *Z. marina* , often in environments that are seasonally stressful for vegetative shoots so that populations persist via seeds (Jarvis and Moore 2015; Keddy and Patriquin 1978; Kim et al. 2014; Meling‐López and Ibarra‐Obando 1999; Morita et al. 2010; Nelson and Sullivan 2018; Phillips et al. 1983; van Katwijk and Tussenbroek 2023; van Lent and Verschuure 1994). Annual ecotypes in the temperate northeast Pacific have recently been shown to be under genetic control (Briones Ortiz et al. 2025). Because annual ecotypes reliably flower, their phenology can be used to assess sensitivity and response to temperature, including population‐specific responses, given that perennial *Z. marina* shows evidence of local adaptation to thermal regimes (DuBois et al. 2022). We use a common garden approach to experimentally test the effect of +3°C elevated seawater temperature on *Z. marina PEBP* gene (*ZmaPEBP*) expression and individual and population‐level flowering traits. We predicted that:

1. Flowering onset would occur earlier in the +3°C treatment and could influence the timing and amount of seed production.
2. Treatments with earlier or more frequent flowering would have lower expression of the antiflorigen repressor of flowering (*ZmaFT9*) and higher expression of activators (*ZmaFT2* and *ZmaFT4*).
3. Natural variation in populations likely plays a role in the response of flowering to environmental cues.

These traits are directly linked to plant fitness and population resilience of these aquatic foundation species.

## 2 Materials and Methods

### 2.1 Field Sites and Collection

**FIGURE 1:**
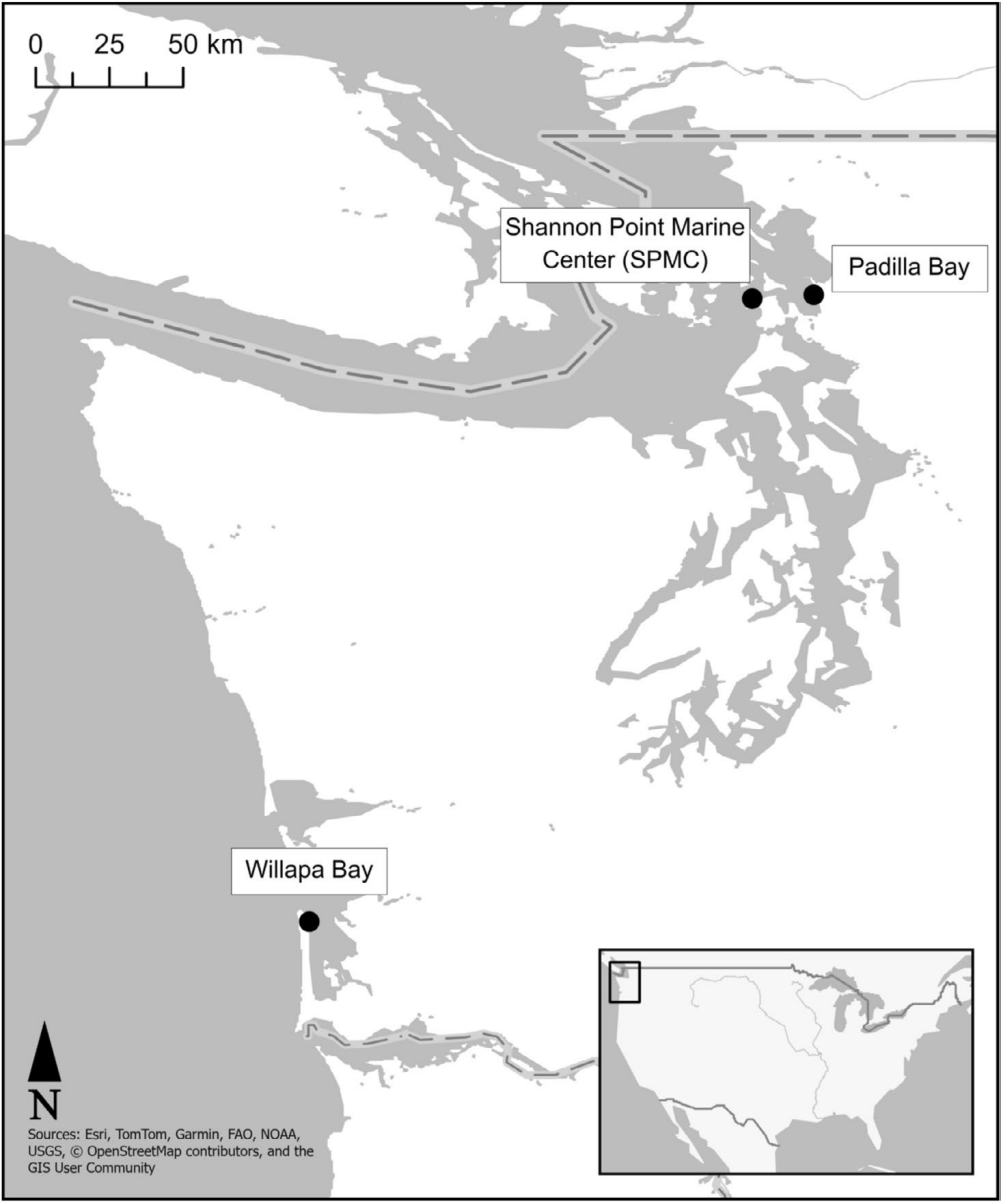
Map of source populations used in the study (Willapa Bay and Padilla Bay). The mesocosm facility is also shown (Shannon Point Marine Center).

**FIGURE 2:**
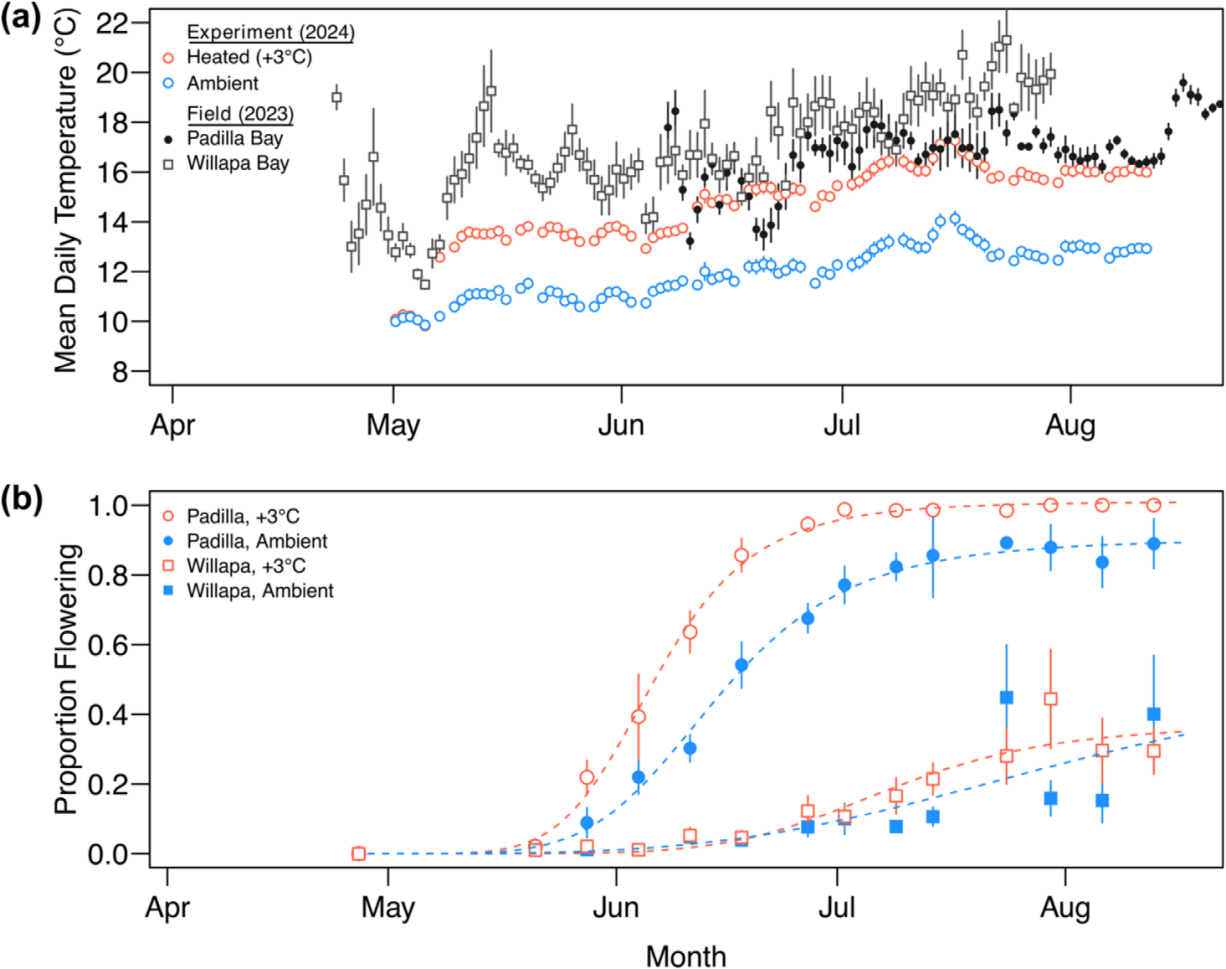
Field and experimental temperature regimes and flowering proportion in the mesocosm set‐up. (a) Mean daily temperatures (mean ± S.E.) at each field collection site in 2023 and in the experimental tanks over the duration of the common garden experiment in 2024. Field sites are intertidal, so temperatures are a combination of water and air temperatures. (b) Proportion of seedlings flowering over the duration of the experiment. Dashed lines show the log‐logistic model fit for each combination of population (Willapa or Padilla Bay) and temperature treatment (+3°C or ambient).

To investigate potential population variation in flowering mechanisms, we collected *Z. marina* seedlings from two locations in Washington state (USA) where annuals are known to be found, Padilla and Willapa Bays (Figure 1, Table S1). Willapa Bay (WB) is located on the coast of Washington State and is estuarine, whereas Padilla Bay (PB) is in the central Salish Sea and has a highly modified shoreline and is orphaned from riverine influences. This difference in geographic location leads to differences in temperature and tidal regimes (Figure 2a).

*Z. marina* seedlings were collected April 26, 2024, from intertidal locations where annual life history has previously been found in the two embayments, co‐occurring with perennial *Z. marina* (Table S1). Thus, seedlings could not be identified as annual or perennial until they flowered during the experiment. Seedlings were carefully extracted from the sediment to preserve roots and were soaked for 2–4 h in < 10 ppt seawater to reduce transfer of *Labyrinthula* sp. and other hitchhikers.

### 2.2 Experimental Design

Within 8–24 h of collection, *Z. marina* seedlings from each bay were planted into eight seawater mesocosms at the Shannon Point Marine Center (SPMC, Table S1, Figure 1). Breiter et al. (2024) provided a detailed description and image of the system, in which seawater was continuously piped into the eight mesocosms from an offshore source (Guemes Channel) with a flow rate of 10 L/min and continuously discharged back into the Guemes Channel.

To experimentally test the effect of elevated seawater temperature on seedling performance and flowering metrics, four of the mesocosms were equipped with custom‐built heat exchangers (Breiter et al. 2024) to elevate seawater temperature conditions by +3°C (+2.73°C ± 0.02°C [mean ± s.e.]). The heating system was designed to achieve the +3°C increase, while preserving natural (ambient) diel and seasonal temperature fluctuations (Figure 2a, see Breiter et al. 2024). We chose a +3°C temperature increase based on predicted global warming trends (Intergovernmental Panel on Climate Change [IPCC] 2023) and predicted warming trends of the Salish Sea (Khangaonkar et al. 2019; Mote and Salathé 2010). The other four remaining mesocosms experienced unmanipulated (unheated), ambient seawater temperature conditions to serve as a basis of comparison to the heated mesocosms.

In these eight mesocosms (4 heated and 4 unmanipulated ambient mesocosms), the *Z. marina* seedlings collected from the two field sites were rooted into replicate tubs of masonry sand that had previously been conditioned for 1 week in the mesocosms (32 seedlings per tub, 51 cm L × 38 cm W × 10 cm sediment depth). Two tubs, one for each source population, were placed in each of the eight seawater mesocosms. Seedlings were reared continuously in the heated (*n* = 4) and ambient (*n* = 4) mesocosm conditions for approximately 3.6 months (108 days) to observe the effects of increased seawater temperature on phenology, quantity, and morphology of seedlings as they developed into flowering shoots.

### 2.3 Population and Morphological Traits

Every week, seedlings were counted in each tub (from each heated and ambient mesocosm) as nonflowering or flowering, and every other week, morphological measurements were recorded: shoot length, number of leaves, and leaf width for 3 haphazardly selected non‐flowering seedlings (if present); and flowering shoot length, number of spathes, and developmental stage of each spathe (De Cock 1980) for 3 flowering seedlings per tub replicate (if present). Seedlings were considered “flowering” as soon as they began to bolt. At the end of the experiment, all flowering shoots were scored for spathe developmental stage. Below‐ground parameters were obtained from biweekly destructive sampling, described below.

### 2.4 *ZmaPEBP* Gene Expression and Individual Traits

Two seedlings from each population's replicate tub were destructively sampled from each mesocosm for gene expression analysis on a weekly basis from 2024‐05‐06 until 2024‐06‐05, and on a biweekly basis after seedlings had flowered (between 2024‐06‐05 and 2024‐07‐29), aiming to capture the window in which flowering is induced. Plants were collected from the mesocosm tubs with roots and rhizomes intact, between ZT3‐5 (Zeitgeber time: hours from sunrise) (Song et al. 2018). All collected seedlings were stored in RNAlater stabilization solution (ThermoFisher, Waltham, MA) and kept at −20°C until processing, at which point individual morphological measurements were taken for shoot length, number of leaves or spathes, leaf width, number of branches, rhizome length, number of rhizome internodes, and root length, as well as developmental state (vegetative, bolting, and flowering). The number of internodes at the beginning of the experiment was used as a proxy to compare age at time of collection from the two source populations. Tissue was frozen at −80°C for long‐term storage.

Gene expression analysis was conducted in the manner as described in Nolan et al. (2025). Total RNA was extracted with the RNeasy Plant Mini Kit (Qiagen, Hilden, Germany) with an on‐column DNA digestion incubation. Complementary DNA (cDNA) was made with the iScript cDNA synthesis kit (Bio‐Rad, Hercules, CA). Locus‐specific primers to detect expression of *ZmaFT2*, *ZmaFT4*, *ZmaFT9*, and *ZmaTFL1a* are described in Nolan et al. (Table S2; Nolan et al. 2025). For reference genes, locus‐specific primers to detect expression of *CYCLOPHILIN 2* (*CYP2*), *EUKARYOTIC INITIATION FACTOR4A* (*ELF4A*), and *RIBOSOME STRUCTURAL PROTEIN L28* (*RPL28*) are described in Ransbotyn and Reusch (Ransbotyn and Reusch 2006). For all qPCR runs, we set a baseline threshold of relative fluorescence units (RFU) for consistency across primers and to ensure that plates were comparable. Samples with no detectable expression were given a cycle threshold (*C*_T_) value of 40 (*C*_T_ = 40). *C*_T_ values were analyzed using the Δ*C*_T_ method, and average *C*_T_ values for the three reference genes were used for calculation.

### 2.5 Statistical Analyses

We aimed to quantify differences in the morphological traits between the two populations (WB and PB) and the two treatments (ambient and +3°C heated) to capture the effects of increased temperature on growth, reproductive timing, and variability of response by population.

To estimate differences in flowering timing, the proportion of flowering was fit to a 3‐parameter log‐logistic model using the *drm* function in R (Ritz et al. 2015). Proportion flowering was calculated per tub from the number of flowering shoots divided by the total number of remaining shoots at each time point. The location parameter *e* (the time to reach 50% of total flowering) was used to estimate relative differences in flowering time between populations and temperature treatments. The parameter *d* was used to assess the fraction of seedlings that were annual and flowered (versus pre‐flowering annuals or perennials that would not be expected to flower during the experiment). The parameter *b* was used to represent the synchronicity of the flowering event. The *compParm* function was used to compare model coefficients among treatment–population combinations.

To test the effect of population (WB and PB) and temperature treatment (ambient or +3°C heated) on morphological traits, 3‐way mixed effects ANOVAs were conducted using the *aov* function in R (R Core Team 2021) with the following model: response ~ treatment * population * census date as fixed effects and tank as a random effect to account for multiple measurements of the same mesocosms over time. Date was considered a categorical variable. Morphological traits of the nonflowering or flowering seedlings were averaged by population and mesocosm replicate prior to analysis. For the final spathe developmental stage and initial and final rhizome internode counts, individual data were analyzed with a 2‐way ANOVA using population * treatment as fixed effects. Post hoc pairwise comparisons were conducted using the *emmeans_test* function (Searle et al. 1980) with Bonferroni correction to account for multiple testing. Post hoc comparisons were also conducted using the Holm–Bonferroni method, which produced slightly different adjusted *p*‐value output values but provided the same result in terms of statistically significant pairwise comparisons.

We used a generalized additive mixed effect model (GAMM) to analyze the expression of *ZmaPEBP* genes across time, since GAMMs account for the non‐unidirectional response of gene expression over time. We used gene expression values as response variables and temperature treatment and population as main effects. Date (continuous) was included as a global thin‐plate regression spline smooth term. As an additional smooth term, the interaction between date and treatment was included to account for possible changes in the timing of expression. Tank number was also included as a random effect smooth term since two individuals were sampled per mesocosm on each date. Expression values were log10‐transformed to fit the assumptions of the model. Statistical models were built in R (R Core Team 2024) using the *gam* function from the package *mgcv* (Wood 2011).

## 3 Results

### 3.1 Population and Morphological Traits

#### 3.1.1 Flowering Timing

A higher proportion of seedlings from Padilla Bay flowered under +3°C heated conditions, but temperature treatment did not have an effect on the proportion of flowering seedlings from Willapa Bay over the duration of the experiment. Annual seedlings from Padilla Bay flowered approximately 9 days earlier in the heated treatment compared to ambient, and 50% of the seedlings in the heated treatment from Padilla Bay flowered approximately 30 days before that of seedlings from Willapa Bay (Figure 2b, Table S3 parameter *e*). Nearly all seedlings from Padilla Bay flowered in heated tanks compared to 90% of seedlings in ambient tanks, which was greater than Willapa Bay seedlings (40%–60%), which were not affected by temperature treatment (Figure 2b, Table S3 parameter *d*). Willapa Bay's maximum flowering was probably reduced by contamination from perennial seedlings. Synchronicity of flowering (parameter *b*) did not differ among temperature treatments or populations.

#### 3.1.2 Morphological Traits

**FIGURE 3:**
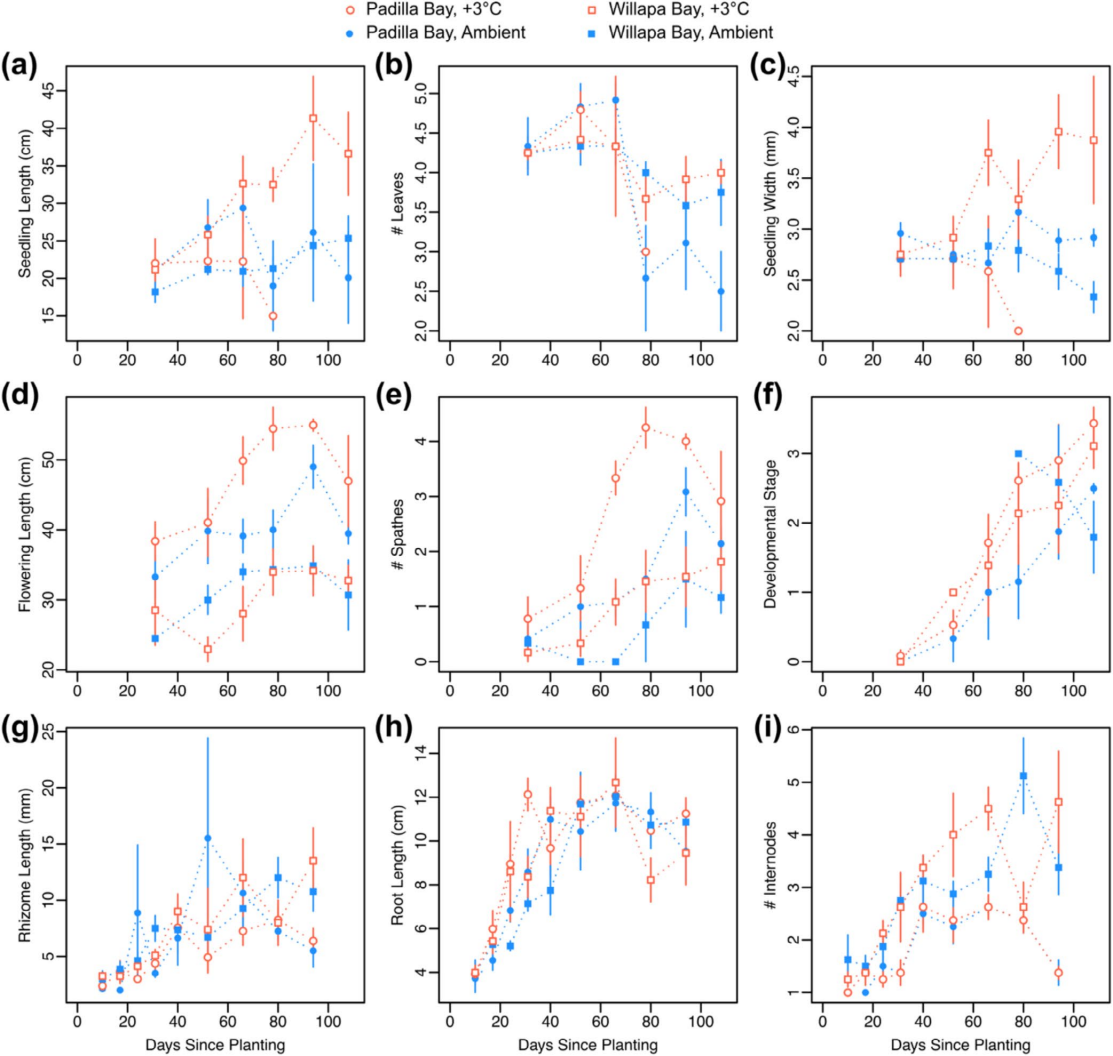
Mean above‐ground seedling morphology prior to flowering (a–c): Nonflowering seedling length, number of leaves, and leaf width; and for shoots with flowers (d–f): Flowering shoot length, mean number of spathes per flowering shoot, and mean developmental stage of spathes per flowering shoot. Mean below‐ground seedling morphology (g–i): Rhizome length, root length, and number of rhizome internodes. For below‐ground measurements, flowering and non‐flowering seedlings are combined. All seedlings were planted on April 26, 2024. Symbology: PB = Padilla Bay (circle); WB = Willapa Bay (square); H = heated (open, red); A = ambient (filled, blue).

Nonflowering seedling length and width increased over time prior to flowering, but did not differ between treatments or populations (Figure 3a,b, Table S4). Nonflowering seedlings were primarily limited to the Willapa population by the end of the experiment since the majority of Padilla seedlings flowered by day 80 (Figure 3c). The width of leaves on these nonflowering seedlings increased over time due to treatment (after day 94) (Figure 3c, Table S4).

For flowering seedlings, seedling length increased over time but did not differ by temperature treatment (Figure 3d, Table S4). The number of spathes on flowering shoots increased over time and differed between populations and temperature treatment. Flowering seedlings in the heated treatment produced 0.8 more spathes than those in the ambient treatment (Figure 3e, Table S4). However, population differences became apparent. Specifically, flowering seedlings from Padilla Bay showed longer leaf length regardless of temperature treatment and produced 1.2 more spathes than those from Willapa Bay (Figure 3d,e, Table S4).

At the end of the experiment (day 108), the spathe developmental stage was 0.9 stages further along in the heated treatment for both populations compared to ambient, and Padilla Bay spathes were 0.4 developmental stages ahead of Willapa Bay spathes (Figure 3f, Table S4). Peak pollination also occurred approximately 2 weeks earlier in heated tanks (Figure S1).

Rhizome and root length of the seedlings also increased over time (flowering and nonflowering seedlings combined) but did not differ by treatment or population (Figure 3g,h, Table S4). The number of internodes did not differ between populations or treatments at the beginning of the experiment. By the end of the experiment, the number of rhizome internodes exhibited a significant site effect, with post hoc pairwise comparisons demonstrating Willapa seedlings having more internodes than Padilla seedlings over time (Figure 3i, Table S4). By the last destructive sampling (day 94), Willapa Bay plants had 2.6 more internodes than Padilla Bay plants (Figure 3i, Table S4). Thus, temperature treatment did not have an effect on rhizome length or rate of internode production, but internode production rate was different between populations.

### 3.2 Gene Expression

**FIGURE 4:**
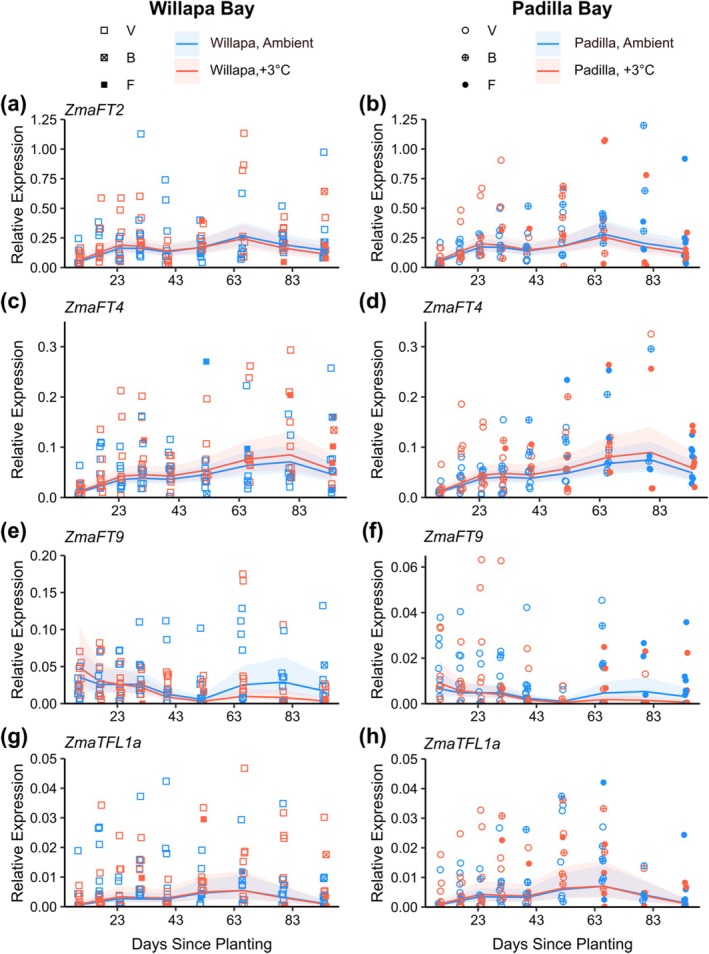
Relative expression of *ZmaFT2* (a, b), *ZmaFT4* (c, d), *ZmaFT9* (e, f), and *ZmaTFL1a* (g, h) genes in leaf tissue during annual growing season (May–July, shown as days since planting, which took place 2024‐04‐26) from both populations (Willapa, square, left; Padilla, circle right) within the mesocosm, plotted with predicted expression trends based on the general additive mixed effect model (GAMM). Blue lines represent ambient‐treated samples, and red lines indicate +3°C treated samples, and the respective ribbons indicate confidence interval. Point shape represents the development state at the time of collection; vegetative (V), bolted (B), flowering (F). Data points above respective axis limits not shown to better visualize model fit, but included in model analysis (*ZmaFT2*, 1.25; *ZmaFT4*, 0.35; *ZmaFT9*, 0.2 for WB and 0.065 for PB; and *ZmaTFL1a*, 0.5). All expression values are relative to 3 reference genes (*CYP2*, *ELF4A*, and *RPL28*).

To gain insight into how sustained increased temperature affects the genes involved in eelgrass flowering, we characterized expression of *ZmaFT2*, *ZmaFT4*, *ZmaFT9*, and *ZmaTFL1a* at various time points during the annual shoot's lifespan in both populations. *ZmaFT2* and *ZmaFT4* showed increased expression over time in both Padilla Bay and Willapa populations (Figures 4a–d, S2a–d and Table S5). *ZmaFT2* expression peaked in early July (2024‐07‐01), and *ZmaFT4* expression peaked 2 weeks after at the following collection (2024‐07‐15). Gene expression patterns in both genes did not differ between temperature treatments or between source populations. Expression of *ZmaFT9* was lower in the +3°C treatment and was lower in shoots from Padilla Bay (Figures 4e,f, S2e,f), which flowered earlier compared to Willapa Bay (Figure 2b). In Padilla Bay samples, *ZmaFT9* showed higher levels of expression in earlier stages of vegetative development and an overall decrease before and after flowering onset. *ZmaFT9* expression decreased approximately 7 days earlier in the +3°C heated treatment compared to the ambient treatment (2024‐05‐20 and 2024‐05‐27, respectively) (Figure 4f), which may contribute to the significant interaction effect between temperature treatment and date in the GAMM results (Table S5). *ZmaTFL1a* expression, in both populations, showed no temporal trend or change due to temperature treatment (Figure 4g,h).

## 4 Discussion

In this study, we characterized the effects of elevated temperature on *Z. marina* flowering from *ZmaPEBP* gene expression (*ZmaFT* and *ZmaTFL1a*) *to* functional ecological traits. We observed earlier flowering and an acceleration of spathe development in flowering shoots from Padilla Bay in the +3°C treatment. Among the four *ZmaPEBP* genes we analyzed, only the antiflorigen gene, *ZmaFT9*, was affected by elevated temperature. These results suggest that *ZmaFT9* may be a major determinant of when flowering onset occurs under different temperatures in *Z. marina*.

### 4.1 Elevated Temperatures Result in Earlier Flowering in Eelgrass Annuals

Our results support the hypothesis that elevated temperatures accelerate the timing of flowering and influence seed production. Thus, the annual life history of *Z. marina* aligns with previous studies in perennials showing earlier flowering processes at warmer temperatures, both artificially manipulated in mesocosms and naturally occurring over a latitudinal gradient (Blok et al. 2018; Sawall et al. 2021). The latitudinal gradient involves a shift of −12.3 day per +1°C and a longer duration of flowering at warmer locations (Blok et al. 2018), whereas we found a −9 day shift per +3°C within a population.

In this study, the acceleration in flowering under warming was also associated with greater production of spathes and quicker development of seeds (Figure S1). Spathe development was further accelerated by an additional week in the +3°C treatment, given that seed dispersal occurred in flowering shoots 2 weeks earlier compared to ambient conditions (Figure S1). Additionally, seeds (from Padilla Bay) took at least 60 days to mature under heated mesocosm conditions after the first onset of styles erecting (13°C–16°C, Figures 2, S1), as compared to De Cock (De Cock 1980), who found the seed maturation process for *Z. marina* from the Netherlands to take 33 days at 20°C.

These collective findings suggest that, with warming, annual seedlings may produce earlier and potentially higher seed yields if the number of spathes produced can act as a proxy for potential seed production. The level of warming in the mesocosms had not apparently reached a stressful level, identified for terrestrial annual crops to warming, where earlier flowering led to lower seed yield (Bassu et al. 2021; Ullah et al. 2022). Warm years have also been linked to lower flowering rates in some populations of perennial *Z. marina* (Qin et al. 2020; Thom et al. 2003). However, the number of seeds per spathe under elevated temperatures remains to be quantified for annual *Z. marina* , as mesocosm conditions were not expected to lead to natural pollination and seed set for estimating seed yield in this experiment.

### 4.2 *ZmaFT9* Expression Is Decreased in Elevated Temperature and May Be Determinant of Earlier Flowering in Warmer Conditions

*PEBP* genes that control flowering onset are largely regulated by temperature, among other stimuli. In the model plant *Arabidopsis*, *FT* gene expression is activated by increased temperature (Balasubramanian et al. 2006; Blázquez et al. 2003; Kinmonth‐Schultz et al. 2016; Susila et al. 2021). Given that eelgrass flowering occurs earlier under warmer conditions (Blok et al. 2018; Sawall et al. 2021), we would expect that *ZmaFT* homologs are similarly regulated by temperature and that activators of flowering would experience an increase in expression in heated conditions. However, in the four eelgrass *PEBP* genes that we tested, only *ZmaFT9* expression, the antiflorigen gene with apparent repressive function, was affected by the +3°C treatment. *ZmaFT9* was previously described to be a repressor of flowering and speculated to be the main determinant of flowering in eelgrass due to a decrease in expression occurring prior to flowering onset (Nolan et al. 2025). Shoots grown in the heated treatment showed lower expression of *ZmaFT9* throughout the entire season in both populations. The decrease in expression over time, which is expected to precede the onset of flowering, seems to occur earlier in the Padilla Bay population in the +3°C treatment compared to the ambient, by approximately 7 days, though we were unable to resolve this difference statistically given the variation within our datasets. Nonetheless, temperature is a key factor in the regulation of *ZmaFT9* expression.

Expression of *ZmaFT2* and *ZmaFT4*, previously described as flowering activators (Nolan et al. 2025), was unaffected by the temperature treatment. While our findings confirm that expression of *ZmaFT2* and *ZmaFT4* increases over time as the plant develops, we observed no significant difference in expression between treatments, contradicting our original hypothesis. Our finding that *ZmaFT2* and *ZmaFT4* are seemingly unregulated by temperature is unexpected, given the effects of increased temperature on florigen in other angiosperms (Balasubramanian et al. 2006; Blázquez et al. 2003; Kinmonth‐Schultz et al. 2016). However, it is not entirely novel for flowering‐related gene expression to be regulated independently of temperature. The Cape Verde Islands' accession of *Arabidopsis* is temperature insensitive due to a hyperactive gain‐of‐function allele in *CYP2* (*CRY2*) (Liu et al. 2013; Sanchez‐Bermejo et al. 2015). Also, most studies on the temperature‐dependent regulation of *FT* are conducted under constant temperature conditions, rather than elevated temperatures relative to the ambient environment. Constant conditions may alter the diurnal regulation of *FT* genes, which have been shown to be affected by standard light regimens within artificial conditions (Song et al. 2018). Given our results, we can conclude that *Z. marina* flowering includes a mechanism for incorporating temperature signals involving *ZmaFT9* regulation but not *ZmaFT2* and *ZmaFT4*. We speculate that the thermal insensitivity of *ZmaFT2* and *ZmaFT4* may reflect *Zostera*'s adaptation to a highly variable intertidal thermal regime, thereby preventing precocious flowering.

### 4.3 Natural Variation in Populations Affects Flowering Response and Related Morphology

Beyond the difference in flowering morphology, development, and gene expression because of the +3°C treatment, we also observed differences in flowering traits that were attributed to natural variation between populations. Compared to Willapa Bay seedlings, annuals from Padilla Bay were longer, produced more spathes (Figure 3d,e, Table S4), and showed overall lower expression of *ZmaFT9* (Figure 4e,f, Table S5). In field collections of annuals from Willapa Bay, *ZmaFT9* expression was relatively high and then fell prior to flowering (Nolan et al. 2025), but no mesocosm samples showed these initially high levels (mostly < 0.05 relative expression; Figure 4). Therefore, population‐specific expression of *ZmaFT9* could be from genetic differences associated with geographic separation (Duffy et al. 2022), different representation of annual ecotypes, or RNA sampling that missed early and high expression of antiflorigen. These three explanations (population genetics, representation by annual ecotypes, and timing of sampling relative to life history) could also apply to morphological trait differences between populations (Figure 3).

Local adaptation can contribute to differential responses to environmental stimuli (Lasky et al. 2014), and our two populations from a similar latitude eliminate likely adaptation across photoperiod. The source sites experience different thermal conditions, with overall higher and more varied temperatures at Willapa Bay (Figure 2a). Temperatures in the mesocosms were lower than summer field temperatures at the source sites (Figure 2), though heated conditions approached natural water temperatures for Padilla Bay, providing another possible explanation for population differences found in this study if local adaptation is at play. A major population‐level difference was in flowering rates, and it seems likely that the lower flowering for Willapa Bay was due to the collection of some perennial seedlings along with annuals. Of the shoots analyzed for gene expression in the final two sampling periods, 30 of 32 were flowering, and the remaining two had bolted from Padilla Bay, but only 4 bolted and 6 flowered from Willapa Bay (Figure 4).

Additionally, other environmental conditions may affect flowering in annual *Z. marina* that have not been addressed by this study. Both annual source populations came from intertidal sites (Table S1), with Willapa Bay plants coming from an elevation near the upper limit observed for the species in Washington state (Christiaen et al. 2022). Yet plants in our study remained submerged throughout the experiment in order to focus on the effect of water temperature. However, water temperature may interact with or be outweighed by desiccation stress to drive flowering and the occurrence of annuals in the species' upper elevational range (Boese and Robbins 2008; Harrison 1993).

It is also possible that seedlings could have been compromised by transplant shock, so differences between treatments should be considered relative. Nonetheless, it is clear that elevated seawater temperature accelerates sexual reproduction through genetic mechanisms, likely including *ZmaFT9* expression and function. Further study will be needed to evaluate the effects of warming on flowering abundance and dynamics in perennial *Z. marina* . Additionally, continued study is needed to understand the effect of warming on germination and seedling performance of annual and perennial *Z. marina* in order to make extensions to long‐term population dynamics and recovery potential following disturbance.

### 4.4 Implications

Our findings present mechanistic insight into how eelgrass populations may respond to warming temperatures predicted to occur in Washington state over the next 100 years (Khangaonkar et al. 2019; Mote and Salathé 2010). From this study, the response of flowering under warming appears to be derived at the level of antiflorigen gene expression with contributions from population‐level variation. The plasticity in *ZmaFT9* gene expression and downstream impacts on flowering development and seed yield have potential ecological consequences, especially in areas where annual and perennial life history types coexist. Persistence of the *Z. marina* seagrass species to warming waters may rely on shifts in life history, such as in enhanced flowering (mixed‐annual‐like histories), essential to maintaining *Z. marina* at its southern range limit in the western Atlantic (Jarvis et al. 2012).

Globally, seagrasses are already experiencing decline rates of greater than 7% per year, threatening the persistence of coastal ecosystems reliant on seagrass ecosystem services (Orth et al. 2006; Waycott et al. 2009). Seeds have shown promise as a tool for restoration of eelgrass populations (Jarvis et al. 2014; Kendrick et al. 2012; Marion and Orth 2010; Orth et al. 2012, 2020; van Katwijk et al. 2016). However, not all populations flower and produce seeds at equal rates, and flowering rates can be especially low in perennial populations (Ruesink et al. 2022; Yang et al. 2013). Continued research is needed on the genetic and epigenetic bases for flowering time, as well as factors influencing seed production and quantity, to address population‐level vulnerability, effective regional conservation, and adaptive significance of traits (Oh et al. 2019). Collectively, this work highlights the need for continued mechanistic investigation into reproductive responses of seagrass to changing environmental conditions, including but not limited to the current study's focus on flowering and seed development. These efforts will also give insight into how to engage this gene‐to‐trait variability in support of the resiliency and persistence of these marine foundation species.

## Author Contributions

**Christine T. Nolan:** conceptualization (equal), data curation (equal), formal analysis (equal), funding acquisition (equal), investigation (equal), methodology (equal), project administration (equal), validation (equal), visualization (equal), writing – original draft (equal), writing – review and editing (equal). **Ian T. McBride:** conceptualization (equal), data curation (equal), formal analysis (equal), investigation (equal), methodology (equal), validation (equal), visualization (equal), writing – original draft (equal), writing – review and editing (equal). **Niyah Reid:** data curation (equal), formal analysis (equal), investigation (equal), visualization (equal). **Sylvia Yang:** conceptualization (equal), data curation (equal), formal analysis (equal), funding acquisition (equal), investigation (equal), methodology (equal), project administration (equal), resources (equal), supervision (equal), validation (equal), visualization (equal), writing – original draft (equal), writing – review and editing (equal). **Takato Imaizumi:** conceptualization (equal), investigation (equal), methodology (equal), resources (equal), supervision (equal), writing – review and editing (equal). **Jennifer L. Ruesink:** conceptualization (equal), formal analysis (equal), investigation (equal), methodology (equal), resources (equal), supervision (equal), writing – review and editing (equal). **Jeffrey L. Gaeckle:** conceptualization (equal), funding acquisition (equal), resources (equal), writing – review and editing (equal).

## Funding

This research was funded by Washington State Department of Natural Resources interagency agreements (93‐106455 to T.I. and 93‐102512 awarded to S.Y.) and U.S. Geological Survey Northwest Climate Science Adaptation Center award G17AC00218 to C.T.N.

## Conflicts of Interest

The authors declare no conflicts of interest.

## Supporting information


**Appendix S1:** ece372942‐sup‐0001‐AppendixS1.docx.

## Data Availability

All data and code are available on Dryad at the following link (https://doi.org/10.5061/dryad.612jm64hd).
