# Supplementary material for: Accelerated Flowering and Differential Florigen Gene Expression of Seagrass Zostera marina Under Experimental Warming
Source: Ecol Evol. 2026 Jan 14;16(1):e72942. doi: 10.1002/ece3.72942 (PMC12801141; doi:10.1002/ece3.72942)
Supplement: Supplementary file 1 — Appendix S1: ece372942‐sup‐0001‐AppendixS1.docx. [file ECE3-16-e72942-s001.docx]

**Supplemental Information**

**Table S1:** GPS coordinates and tidal elevation of study sites

**Table S2:** qPCR primers used in this study for gene expression analysis

**Table S3:** Log-logistic model parameter estimates for time to reach 50% (a) and pairwise comparisons (b)

**Table S4:** Results from mixed-effects ANOVA tests for morphological measurements

**Table S5:** Generalized additive model (GAM) results with gene expression values as response variable and temperature treatment (Ambient or +3°C Heated) and population (Willapa Bay, WB, and Padilla Bay, PB) as main effects.

**Figure S1:** Developmental stage of spathes in each temperature treatment and population over the duration of the experiment

**Figure S2:** Relative expression of *ZmaFT2*, *ZmaFT4*, *ZmaFT9*, and *ZmaTFL1a* genes in leaf tissue during annual growing season

**Table S1**: GPS coordinates and tidal elevation of study sites.

| **Site** | **Abbreviation** | **Position (°N, °W)** | **Tidal Elevation (m MLLW)** |
| --- | --- | --- | --- |
| Stackpole Annual, Willapa Bay WA USA | WB | 46.613, 124.034 | 1.1 ± 0.1 |
| Joe Leary Annual, Padilla Bay WA USA | PB | 48.520, 122.495 | 0.3 |
| Shannon Point Marine Center, Anacortes WA USA (mesocosm facility) | SPMC | 48.508, 122.683 | NA |

**Table S2:** qPCR primers used in this study for gene expression analysis (Nolan et al., 2024; Ransbotyn & Reusch, 2006)

| **Name** | **Accession No.** | **Forward 5’-3’** | **Reverse 5’-3’** | **Reference** |
| --- | --- | --- | --- | --- |
| *ZmaFT2* | Zosma01g14870 | ACACAATGCTCATGGTAGATCCT | AGCACAAACACGAAACGGTG | Nolan et al. |
| *ZmaFT4* | Zosma04g08400 | TGCACTGGTTGGTGGTGAA | AGCCGACTAGGTTGACGGAA | Nolan et al. |
| *ZmaFT9* | Zosma01g13540 | TTCCTACACACTGGTTATGGTCG | GATGGATGGTTGTGGGCTCT | Nolan et al. |
| *ZmaTFL1a* | Zosma04g17290 | TGTTCACTGGATTGTGACAGA | CCAGTGCAAATCGACGTGTG | Nolan et al. |
| *ZmaCyp2* | Zosma05g32090 | CACTCCACTACAAGGGATCGAAA | GGACCTGTATGCTTCTTAACGAAGT | Ransbotyn and Reusch |
| *Zmaelf4a* | Zosma03g02900 | TCTTTCTGCGATGCGAACAG | TGGATGTATCGGCAGAAACG | Ransbotyn and Reusch |
| *ZmaRPL28* | Zosma04g25970 | TTCCGCACCTAGGGTTTCG | ATATTGGCGCAGCGATTTTG | Ransbotyn and Reusch |

**Table S3**: a) Log-logistic model parameter estimates for time to reach 50% flowering (parameter *e*), fraction of seedlings that flowered (parameter *d*), and flowering synchronicity (parameter *b*), by each population in heated versus ambient conditions. b) Pairwise comparisons of parameter estimates for each treatment-population combination.

a)

| **Parameter** | **Site** | **Treatment** | **Estimate** | **Std. Error** | **t-value** | **p-value** |
| --- | --- | --- | --- | --- | --- | --- |
| *b* | PB | ambient | -5.402 | 0.864 | -6.251 | 0.00 |
| *b* | PB | heated | -6.027 | 0.854 | -7.055 | 0.00 |
| *b* | WB | ambient | -3.742 | 2.569 | -1.457 | 0.15 |
| *b* | WB | heated | -6.378 | 3.113 | -2.049 | 0.04 |
| *d* | PB | ambient | 0.903 | 0.036 | 25.417 | 0.00 |
| *d* | PB | heated | 1.010 | 0.025 | 40.305 | 0.00 |
| *d* | WB | ambient | 0.552 | 0.651 | 0.848 | 0.40 |
| *d* | WB | heated | 0.371 | 0.071 | 5.238 | 0.00 |
| *e* | PB | ambient | 48.861 | 1.603 | 30.480 | 0.00 |
| *e* | PB | heated | 40.365 | 1.069 | 37.754 | 0.00 |
| *e* | WB | ambient | 98.948 | 63.099 | 1.568 | 0.12 |
| *e* | WB | heated | 72.207 | 6.182 | 11.680 | 0.00 |

b)

| **Comparison for parameter *d*** | | | | |
| --- | --- | --- | --- | --- |
| **Ratio** | **Estimate** | **Std. Error** | **t-value** | **p-value** |
| PB ambient/PB heated | 0.89 | 0.04 | -2.56 | 0.01 |
| WB ambient/WB heated | 1.49 | 1.78 | 0.27 | 0.78 |
| PB ambient/WB ambient | 1.64 | 1.93 | 0.33 | 0.74 |
| PB heated/WB heated | 2.72 | 0.52 | 3.29 | 0.00 |
| PB ambient/WB heated | 2.43 | 0.47 | 3.02 | <0.01 |
| WB ambient/PB heated | 0.55 | 0.64 | -0.70 | 0.48 |
| **Comparison for parameter *e*** | | | | |
| **Ratio** | **Estimate** | **Std. Error** | **t-value** | **p-value** |
| PB ambient/PB heated | 1.21 | 0.05 | 4.12 | <0.01 |
| WB ambient/WB heated | 1.37 | 0.88 | 0.42 | 0.67 |
| PB ambient/WB ambient | 0.49 | 0.32 | -1.61 | 0.11 |
| PB heated/WB heated | 0.56 | 0.05 | -8.80 | <0.01 |
| PB ambient/WB heated | 0.68 | 0.06 | -5.21 | <0.01 |
| WB ambient/PB heated | 2.45 | 1.56 | 0.93 | 0.35 |
| **Comparison for parameter *b*** | | | | |
| **Ratio** | **Estimate** | **Std. Error** | **t-value** | **p-value** |
| PB ambient/PB heated | 0.90 | 0.19 | -0.54 | 0.59 |
| WB ambient/WB heated | 0.59 | 0.49 | -0.84 | 0.40 |
| PB ambient/WB ambient | 1.44 | 1.02 | 0.44 | 0.66 |
| PB heated/WB heated | 0.94 | 0.48 | -0.11 | 0.91 |
| PB ambient/WB heated | 0.85 | 0.44 | -0.35 | 0.73 |
| WB ambient/PB heated | 0.62 | 0.44 | -0.87 | 0.39 |

**Table S4**: Results from mixed-effects ANOVA tests for morphological measurements, with temperature treatment (‘trt’), site, census date (‘days’), and their interactions as explanatory variables, and with tank replicate as a random effect (repeated measures). Relevant post-hoc pairwise comparisons are shown with p-values adjusted using the Bonferroni correction.

***Non-flowering seedling: Shoot Length (cm)***

Error: tank

|  | Df | Sum Sq. | Mean Sq. | F value | Pr(>F) |
| --- | --- | --- | --- | --- | --- |
| trt | 1 | 543.1 | 543.1 | 5.16 | 0.0528 |
| site | 1 | 151.4 | 151.4 | 1.438 | 0.2647 |
| days | 4 | 953.8 | 238.4 | 2.266 | 0.151 |
| trt:site | 1 | 506.4 | 506.4 | 4.812 | 0.0596 |
| Residuals | 8 | 841.9 | 105.2 |  |  |

Error: Within

|  | Df | Sum Sq. | Mean Sq. | F value | Pr(>F) |
| --- | --- | --- | --- | --- | --- |
| days | 5 | 884 | 176.79 | 5.809 | 0.00032 |
| trt:days | 5 | 285.7 | 57.13 | 1.877 | 0.11722 |
| site:days | 5 | 62.5 | 12.5 | 0.411 | 0.83871 |
| trt:site:days | 3 | 121.8 | 40.61 | 1.334 | 0.27504 |
| Residuals | 45 | 1369.4 | 30.43 |  |  |

***Non-flowering seedling: Number of leaves***

Error: tank

|  | Df | Sum Sq. | Mean Sq. | F value | Pr(>F) |
| --- | --- | --- | --- | --- | --- |
| trt | 1 | 0.485 | 0.4848 | 1.148 | 0.315 |
| site | 1 | 0.13 | 0.1305 | 0.309 | 0.594 |
| days | 4 | 7.769 | 1.9421 | 4.597 | 0.032 |
| trt:site | 1 | 0.085 | 0.0847 | 0.2 | 0.666 |
| Residuals | 8 | 3.38 | 0.4225 |  |  |

Error: Within

|  | Df | Sum Sq. | Mean Sq. | F value | Pr(>F) |
| --- | --- | --- | --- | --- | --- |
| days | 5 | 11.522 | 2.3043 | 6.178 | 0.000194 |
| trt:days | 5 | 1.604 | 0.3207 | 0.86 | 0.515354 |
| site:days | 5 | 3.631 | 0.7263 | 1.947 | 0.105171 |
| trt:site:days | 3 | 0.705 | 0.2351 | 0.63 | 0.599202 |
| Residuals | 45 | 16.784 | 0.373 |  |  |

***Non-flowering seedling: Leaf Width (mm)***

Error: tank

|  | Df | Sum Sq. | Mean Sq. | F value | Pr(>F) |
| --- | --- | --- | --- | --- | --- |
| trt | 1 | 3.246 | 3.246 | 5.071 | 0.0544 |
| site | 1 | 1.005 | 1.005 | 1.57 | 0.2456 |
| days | 4 | 4.522 | 1.131 | 1.766 | 0.2288 |
| trt:site | 1 | 1.714 | 1.714 | 2.678 | 0.1404 |
| Residuals | 8 | 5.122 | 0.64 |  |  |

Error: Within

|  | Df | Sum Sq. | Mean Sq. | F value | Pr(>F) |
| --- | --- | --- | --- | --- | --- |
| days | 5 | 1.169 | 0.2339 | 1.066 | 0.3915 |
| trt:days | 5 | 3.22 | 0.644 | 2.937 | 0.0223 |
| site:days | 5 | 1.7 | 0.3399 | 1.55 | 0.1937 |
| trt:site:days | 3 | 0.73 | 0.2432 | 1.109 | 0.3554 |
| Residuals | 45 | 9.869 | 0.2193 |  |  |

Post-hoc test by treatment (A=ambient, H=heated)

| Days | H-A | df | statistic | p | p.adjusted |
| --- | --- | --- | --- | --- | --- |
| 31 | -0.1 | 67 | 0.369 | 0.713 | 0.713 |
| 52 | 0.08 | 67 | -0.295 | 0.769 | 0.769 |
| 66 | 0.5 | 67 | -1.71 | 0.0915 | 0.0915 |
| 78 | 0.12 | 67 | -0.341 | 0.734 | 0.734 |
| 94 | 1.24 | 67 | -3.52 | 0.000789 | <0.001 |
| 108 | 1.35 | 67 | -3.7 | 0.00044 | <0.001 |

***Flowering seedling: Flowering shoot length (cm)***

Error: tank

|  | Df | Sum Sq. | Mean Sq. | F value | Pr(>F) |
| --- | --- | --- | --- | --- | --- |
| trt | 1 | 148 | 148 | 1.009 | 0.34449 |
| **site** | **1** | **3637** | **3637** | **24.871** | **0.00107** |
| days | 2 | 145 | 73 | 0.497 | 0.6262 |
| trt:site | 1 | 436 | 436 | 2.98 | 0.12258 |
| trt:days | 1 | 59 | 59 | 0.402 | 0.54356 |
| site:days | 1 | 8 | 8 | 0.058 | 0.81562 |
| Residuals | 8 | 1170 | 146 |  |  |

Error: Within

|  | Df | Sum Sq. | Mean Sq. | F value | Pr(>F) |
| --- | --- | --- | --- | --- | --- |
| days | 5 | 1377.7 | 275.55 | 9.055 | <<0.0001 |
| trt:days | 5 | 209.8 | 41.96 | 1.379 | 0.247 |
| site:days | 5 | 140.2 | 28.04 | 0.922 | 0.475 |
| trt:site:days | 5 | 115.4 | 23.08 | 0.758 | 0.584 |
| Residuals | 52 | 1582.4 | 30.43 |  |  |

Post-hoc test by site (PB=Padilla, WB=Willapa)

| PB-WB | df | statistic | p | p.adjusted |
| --- | --- | --- | --- | --- |
| 12.75 | 86 | 7.48 | <<0.0001 | <<0.0001 |

***Flowering seedling: Number of spathes***

Error: tank

|  | Df | Sum Sq. | Mean Sq. | F value | Pr(>F) |
| --- | --- | --- | --- | --- | --- |
| **trt** | **1** | **15.04** | **15.04** | **6.732** | **0.0357** |
| **site** | **1** | **35.34** | **35.34** | **15.816** | **0.00535** |
| days | 3 | 3.64 | 1.21 | 0.542 | 0.66851 |
| trt:site | 1 | 3.07 | 3.07 | 1.373 | 0.27959 |
| trt:days | 1 | 1.92 | 1.92 | 0.86 | 0.3846 |
| site:days | 1 | 1.39 | 1.39 | 0.624 | 0.45544 |
| Residuals | 7 | 15.64 | 2.23 |  |  |

Error: Within

|  | Df | Sum Sq. | Mean Sq. | F value | Pr(>F) |
| --- | --- | --- | --- | --- | --- |
| days | 5 | 50.91 | 10.183 | 14.42 | <<0.0001 |
| trt:days | 5 | 8.42 | 1.684 | 2.385 | 0.0509 |
| site:days | 5 | 5.91 | 1.182 | 1.673 | 0.1579 |
| trt:site:days | 5 | 2.76 | 0.551 | 0.781 | 0.5683 |
| Residuals | 51 | 36.01 | 0.706 |  |  |

Post-hoc test by site (PB=Padilla, WB=Willapa)

| PB-WB | df | statistic | p | p.adjusted |
| --- | --- | --- | --- | --- |
| 1.2 | 85 | 4.33 | <<0.0001 | <<0.0001 |

Post-hoc test by treatment (A=ambient, H=heated)

| H-A | df | statistic | p | p.adjusted |
| --- | --- | --- | --- | --- |
| 0.8 | 85 | -2.78 | 0.00662 | 0.00662 |

***Flowering seedling: Mean developmental stage of spathes (final timepoint only)***

|  | Df | Sum Sq. | Mean Sq. | F value | Pr(>F) |
| --- | --- | --- | --- | --- | --- |
| **trt** | **1** | **21.42** | **21.417** | **16.225** | **0.000108** |
| **site** | **1** | **8.07** | **8.073** | **6.116** | **0.015031** |
| trt:site | 1 | 0.06 | 0.063 | 0.048 | 0.827732 |
| Residuals | 103 | 135.96 | 1.32 |  |  |

Post-hoc test by treatment (A=ambient, H=heated)

| H-A | df | statistic | p | p.adjusted |
| --- | --- | --- | --- | --- |
| 0.9 | 105 | -3.95 | 0.000142 | 0.000142 |

Post-hoc test by site (PB=Padilla, WB=Willapa)

| PB-WB | df | statistic | p | p.adjusted |
| --- | --- | --- | --- | --- |
| 0.4 | 105 | 1.56 | 0.121 | 0.121 |

***Seedling rhizome length (mm, includes both flowering and non-flowering)***

Error: tank

|  | Df | Sum Sq. | Mean Sq. | F value | Pr(>F) |
| --- | --- | --- | --- | --- | --- |
| trt | 1 | 21.1 | 21.12 | 0.513 | 0.487 |
| site | 1 | 48.8 | 48.83 | 1.186 | 0.297 |
| trt:site | 1 | 24.4 | 24.38 | 0.592 | 0.456 |
| Residuals | 12 | 493.8 | 41.15 |  |  |

Error: Within

|  | Df | Sum Sq. | Mean Sq. | F value | Pr(>F) |
| --- | --- | --- | --- | --- | --- |
| days | 8 | 921.5 | 115.19 | 5.599 | <<0.0001 |
| trt:days | 8 | 150.7 | 18.83 | 0.916 | 0.507 |
| site:days | 8 | 218.1 | 27.26 | 1.325 | 0.24 |
| trt:site:days | 8 | 214 | 26.75 | 1.301 | 0.253 |
| Residuals | 96 | 1974.9 | 20.57 |  |  |

***Seedling root length (cm, includes both flowering and non-flowering)***

Error: tank

|  | Df | Sum Sq. | Mean Sq. | F value | Pr(>F) |
| --- | --- | --- | --- | --- | --- |
| trt | 1 | 0.21 | 0.21 | 0.752 | 0.403 |
| **site** | **1** | **42.79** | **42.79** | **153.099** | **<<0.001** |
| trt:site | 1 | 0.04 | 0.04 | 0.155 | 0.7 |
| Residuals | 12 | 3.35 | 0.28 |  |  |

Error: Within

|  | Df | Sum Sq. | Mean Sq. | F value | Pr(>F) |
| --- | --- | --- | --- | --- | --- |
| days | 8 | 80.09 | 10.012 | 16.512 | <<0.001 |
| **trt:days** | **8** | **11.09** | **1.386** | **2.286** | **0.02763** |
| **site:days** | **8** | **16.44** | **2.055** | **3.389** | **0.00181** |
| **trt:site:days** | **8** | **11** | **1.375** | **2.268** | **0.02879** |
| Residuals | 96 | 58.21 | 0.606 |  |  |

Post-hoc tests by trt:days:

| Trt | Days | WB-PB | df | Statistic | p | p.adj |
| --- | --- | --- | --- | --- | --- | --- |
| A | 10 | 0.625 | 108 | -1.17 | 0.24 | 0.24 |
| A | 17 | 0.5 | 108 | -0.94 | 0.35 | 0.35 |
| A | 24 | 0.375 | 108 | -0.70 | 0.48 | 0.48 |
| A | 31 | 1.375 | 108 | -2.58 | 0.01 | **0.01** |
| A | 40 | 0.625 | 108 | -1.17 | 0.24 | 0.24 |
| A | 52 | 0.625 | 108 | -1.17 | 0.24 | 0.24 |
| A | 66 | 0.625 | 108 | -1.17 | 0.24 | 0.24 |
| A | 80 | 2.75 | 108 | -5.15 | 0.00 | **<0.01** |
| A | 94 | 2 | 108 | -3.75 | 0.00 | **<0.01** |
| H | 10 | 0.25 | 108 | -0.47 | 0.64 | 0.64 |
| H | 17 | 0 | 108 | 0.00 | 1.00 | 1.00 |
| H | 24 | 0.875 | 108 | -1.64 | 0.10 | 0.10 |
| H | 31 | 1.25 | 108 | -2.34 | 0.02 | 0.02 |
| H | 40 | 0.75 | 108 | -1.40 | 0.16 | 0.16 |
| H | 52 | 1.625 | 108 | -3.04 | 0.00 | **<0.01** |
| H | 66 | 1.875 | 108 | -3.51 | 0.00 | **<0.01** |
| H | 80 | 0.25 | 108 | -0.47 | 0.64 | 0.64 |
| H | 94 | 3.25 | 108 | -6.09 | 0.00 | **<0.01** |

Post-hoc test by site:days:

| Site | Days | H-A | df | Statistic | p | p.adj |
| --- | --- | --- | --- | --- | --- | --- |
| PB | 10 | 0 | 108 | 0.00 | 1.00 | 1.00 |
| PB | 17 | 0.375 | 108 | -0.70 | 0.48 | 0.48 |
| PB | 24 | -0.25 | 108 | 0.47 | 0.64 | 0.64 |
| PB | 31 | 0 | 108 | 0.00 | 1.00 | 1.00 |
| PB | 40 | 0.125 | 108 | -0.23 | 0.82 | 0.82 |
| PB | 52 | 0.125 | 108 | -0.23 | 0.82 | 0.82 |
| PB | 66 | 0 | 108 | 0.00 | 1.00 | 1.00 |
| PB | 80 | 0 | 108 | 0.00 | 1.00 | 1.00 |
| PB | 94 | 0 | 108 | 0.00 | 1.00 | 1.00 |
| WB | 10 | -0.375 | 108 | 0.70 | 0.48 | 0.48 |
| WB | 17 | -0.125 | 108 | 0.23 | 0.82 | 0.82 |
| WB | 24 | 0.25 | 108 | -0.47 | 0.64 | 0.64 |
| WB | 31 | -0.125 | 108 | 0.23 | 0.82 | 0.82 |
| WB | 40 | 0.25 | 108 | -0.47 | 0.64 | 0.64 |
| WB | 52 | 1.125 | 108 | -2.11 | 0.04 | **0.04** |
| WB | 66 | 1.25 | 108 | -2.34 | 0.02 | **0.02** |
| WB | 80 | -2.5 | 108 | 4.68 | 0.00 | **<0.01** |
| WB | 94 | 1.25 | 108 | -2.34 | 0.02 | **0.02** |

***Initial number of internodes (Day 10)***

|  | Df | Sum Sq. | Mean Sq. | F value | Pr(>F) |
| --- | --- | --- | --- | --- | --- |
| site | 1 | 0.7656 | 0.7656 | 3.128 | 0.102 |
| trt | 1 | 0.1406 | 0.1406 | 0.574 | 0.463 |
| site:trt | 1 | 0.1406 | 0.1406 | 0.574 | 0.463 |
| Residuals | 12 | 2.9375 | 0.2448 |  |  |

***Final number of internodes (Day 94)***

|  | Df | Sum Sq. | Mean Sq. | F value | Pr(>F) |
| --- | --- | --- | --- | --- | --- |
| **site** | **1** | **27.563** | **27.563** | **21.69** | **0.000554** |
| trt | 1 | 1.562 | 1.562 | 1.23 | 0.289231 |
| site:trt | 1 | 1.562 | 1.562 | 1.23 | 0.289231 |
| Residuals | 12 | 15.25 | 1.271 |  |  |

Post-hoc test by site (PB=Padilla, WB=Willapa)

| PB-WB | df | statistic | p | p.adjusted |
| --- | --- | --- | --- | --- |
| -2.6 | 14 | -4.58 | 0.000426 | 0.000426 |

**Table S5:** Generalized additive model (GAM) results with gene expression values as response variable and temperature treatment (Ambient or +3°C Heated) and population (Willapa Bay, WB, and Padilla Bay, PB) as main effects. Estimate is shown for main effects with standard error in parentheses. Date was included as a global thin-plate regression spline (TP) smooth term. The interaction between date and treatment was included as a random factor smooth interactions term (FS). Tank number was included as a random effect smooth term (RE). Expression values were log10 transformed to fit assumptions of model.

|  | ***ZmaFT2*** | ***ZmaFT4*** | ***ZmaFT9*** | ***ZmaTFL1a*** |
| --- | --- | --- | --- | --- |
| **Intercept** | -1.830 (0.090)  t = -16.758  p = <2e-16 | -3.180 (0.110)  t = -28.922  p = <2e-16 | -3.909 (0.196)  t = -19.937  p = <2e-16 | -5.755 (0.223)  t = -28.838  p = <2e-16 |
| **Temperature (Ambient:Heated)** | 0.028 (0.136)  t = 0.204  p = 0.838 | 0.169 (0.137)  t = 1.230  p = 0.220 | -0.484 (0.227)  t = -2.134  p = 0.0337 | 0.0967 (0.283)  t = 0.342  p =0.733 |
| **Population (WB:PB)** | 0.052 (0.105)  t = 0.502  p = 0.616 | 0.056 (0.104)  t = 0.536  p = 0.592 | -1.691 (0.227)  t = -7.445  p =1.32e-12 | 0.252 (0.197)  t = 1.279  p = 0.202 |
| **Date** (TP smoother term) | edf = 5.158  F = 11.624  p = <2e-16 | edf = 5.158  F = 13.833  p =<2e-16 | edf = 5.158  F = 4.090  p = 6.18e-04 | edf = 5.158  F = 7.703  p = 2.48e-07 |
| **Date:Ambient** (FS smooth term) | edf = 0.0002  F = 0.001  p = 1.0 | edf = 1.000  F = 0.005  p = 0.944 | edf = 3.767e-05  F = 0  p = 0.5 | edf = 5.017e-05  F = 0.008  p = 0.999 |
| **Date:Heat** (FS smooth term) | edf = 1.0002  F = 2.076  p =0.151 | edf = 0.000  F = 0.011  p = 0.999 | edf = 1.000  F = 7.435  p = 0.0068 | edf = 1.001  F = 0.425  p = 0.5152 |
| **Tank** (RE smooth term) | edf = 2.556  F = 0.747  p = 0.108 | edf = 2.581  F = 0.763  p = 0.103 | edf = 3.273e-04  F = 0  p = 0.914 | edf = 3.111  F = 1.071  p = 0.0564 |

**
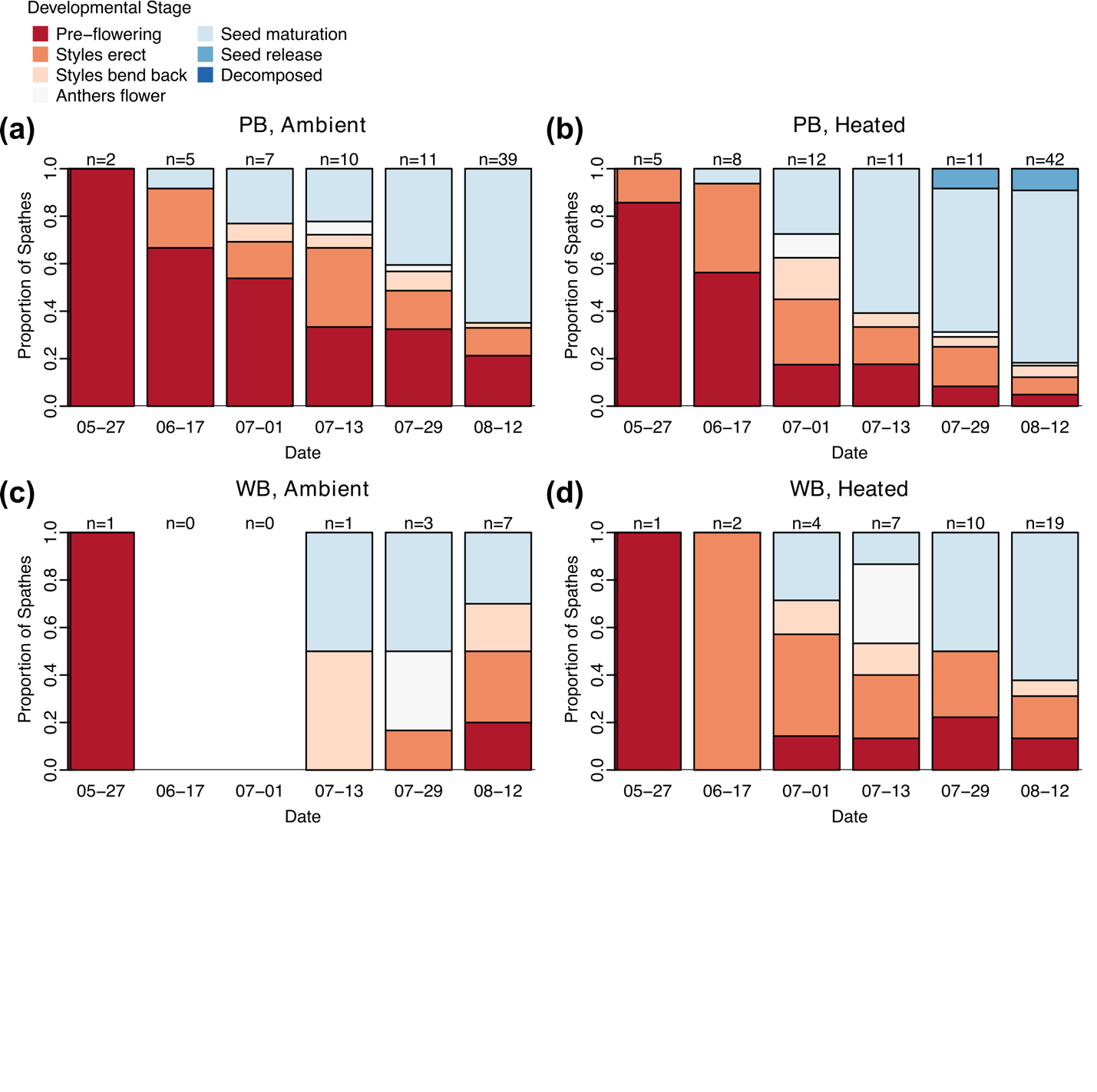
**

**Figure S1:** Developmental stage of spathes in each temperature treatment and population over the duration of the experiment (a-d). Abbreviations: PB = Padilla Bay; WB = Willapa Bay). Sample size shows the number of flowering shoots that were averaged for each census date (mm-dd). Developmental stages are based on De Cock (1980).

**
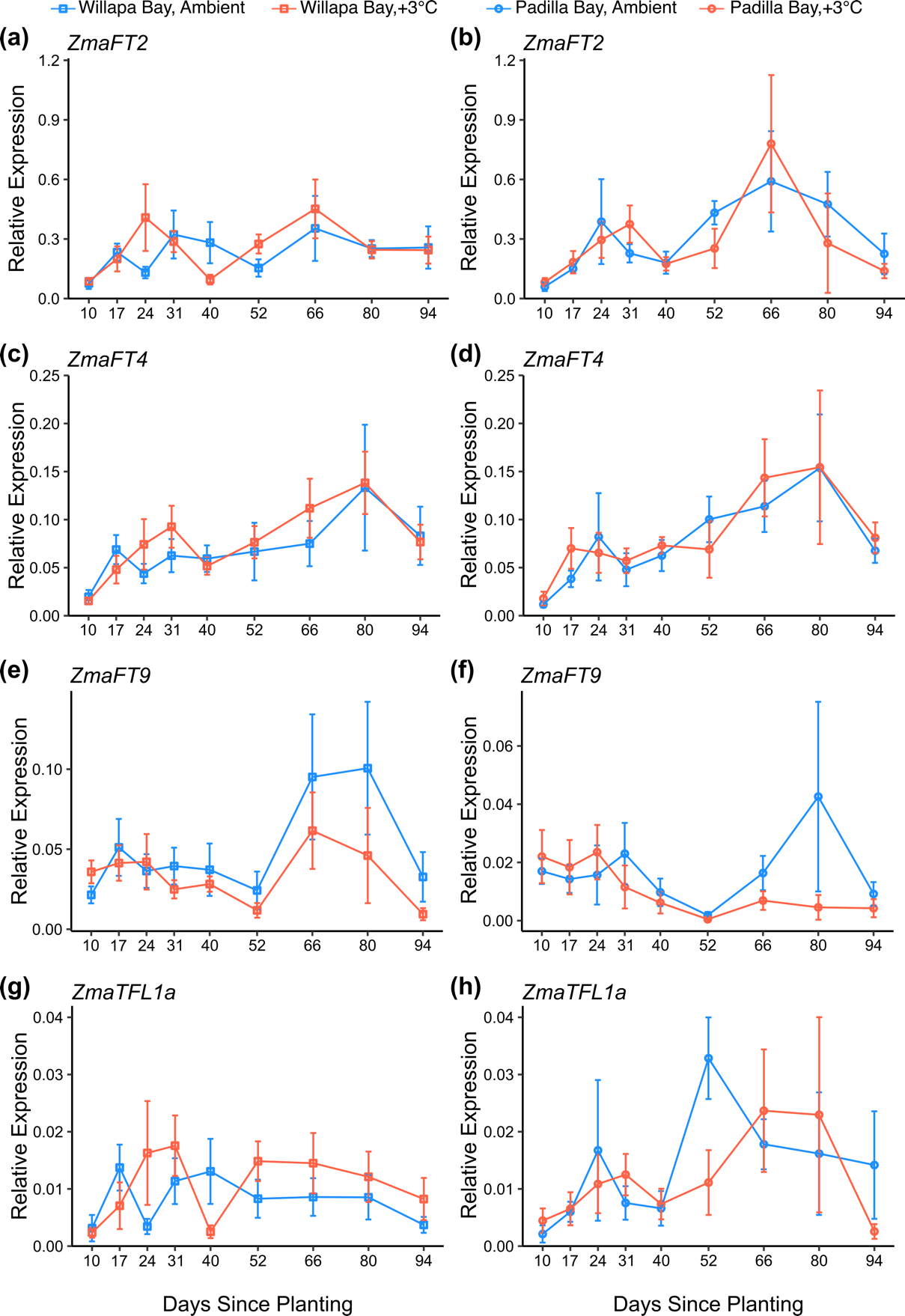
**

**Figure S2:** Relative expression of *ZmaFT2* (a, b), *ZmaFT4* (c, d), *ZmaFT9* (e, f), and *ZmaTFL1a* (g, h) genes in leaf tissue during annual growing season (May-July, shown as days since planting, which took place 2024-04-26) from both populations (Willapa, square, left; Padilla, circle right) within the mesocosm. Plot point represents mean, and error bars are standard error. Blue lines represent ambient treated samples, and red lines indicate +3°C treated samples. All expression values are relative to 3 reference genes (*CYP2*, *ELF4A*, and *RPL28*). Plot point represents mean, and error bars are standard error.
